# The price of possessiveness: how parental materialism undermines child psychological wellbeing

**DOI:** 10.3389/frcha.2025.1600599

**Published:** 2025-08-07

**Authors:** Miao Li

**Affiliations:** Department of Sociology, Anthropology, and Criminal Justice, Clemson University, Clemson, SC, United States

**Keywords:** child, distress, materialism, mental health, parenting, social comparison

## Abstract

Materialism, a value system that places the pursuit of possessions at the core of happiness and life meaning, is a dominant cultural force in modern societies. While its associations with individual well-being are well-documented, its intergenerational implications remain understudied. This study conceptualizes materialism as a potential family stressor contributing to the intergenerational transmission of stress. An intergenerational crossover model of materialism was tested using data from 1,996 parent-child pairs in Zhengzhou, China. Results indicate that higher parental materialism is associated with stronger materialistic values in children, weaker family relationships, and more frequent parental comparisons, each of which is linked to greater psychological distress in youth. These patterns suggest that materialism may contribute to intergenerational patterns of vulnerability. The study highlights the cultural dimensions of mental health and provides a theoretical tool for further research on how materialism, as modernity's “default value”, relates to health inequalities.

[In a Puritan's view], the care for external goods should only lie on the shoulders of the 'saint like a light cloak, which can be thrown aside at any moment'. But fate decreed that the cloak should become an iron cage.

— Max Weber, The Protestant Ethic and the Spirit of Capitalism

## Introduction

The rising tide of youth mental health issues—depression, anxiety, and psychological distress—has been described as a global crisis of modernity (1–4). While often discussed in Western contexts, these trends are increasingly evident in rapidly industrializing societies (5, 6). Dramatic societal changes, urbanization, erosion of traditional communities and values, and the resulting sense of uprootedness—combined with the immense burden of making sense of these changes and adapting to them—are often cited as significant contributors to the growing mental health challenges. Built on existing literature, this study examined an understudied aspect of the cultural dynamic that may underlie the mental health pandemic for future generations. Central to this exploration is the intergenerational mental health impact of a hegemonic modern value system known as “materialism”.

Materialism, broadly defined as the value placed on acquiring material possessions as a path to happiness and life meaning (7–9), reflects a cultural orientation emphasizing “having” over “being”, competition over connection, and acquisition as an end in itself (8–11). Though material desires are not new, modern capitalist societies have increasingly normalized and even enshrined materialism, setting it free from older moral, spiritual, and communal constraints. As Weber (1930/1992) famously observed, the instrumental logic of capitalism transformed a once-“light cloak” into an “iron cage”, entrenching material accumulation as a moral imperative.

While often linked to economic growth and personal ambition, materialism carries well-documented psychological costs. A robust literature finds that individuals high in materialistic values report lower life satisfaction and emotional well-being, along with greater depression, loneliness, anxiety, and addictive behaviors (7, 12–18). Materialism is also associated with reduced prosocial behavior, weaker environmental concern, and strained interpersonal relationships (19–23).

Sociological theorists have long warned of these broader harms. From Marx's critique of “commodity fetishism” to Bauman's diagnosis of “liquid consumerism”, scholars have argued that materialism promotes objectification, alienation, and ultimately psychological deprivation. Yet most empirical research focuses on individual-level outcomes, with little attention to how materialistic values might affect others, especially across generations.

This study addresses that gap by proposing and testing an intergenerational crossover model. Drawing on data from over 2,000 parent-child dyads in a fast-growing Chinese city, I examine whether and how parental materialism predicts child psychological distress through three mechanisms: (1) value transmission, (2) deterioration in relationship quality, and (3) increased parental social comparison of the child. Together, these pathways reflect how culturally dominant value systems can shape mental health through relational processes within families.

## Background

### The rise of materialism in China

China's rapid economic growth and dramatic social transformation over recent decades provide a revealing context for examining the generational impacts of materialism. Since the communist regime's inception in 1949, China has undergone an accelerated process of modernization, accomplishing in decades what took centuries in the West. A confluence of historical events has fostered the emergence of a pervasive materialistic culture in contemporary China.

The communist regime's radical movements of the 1950s dismantled traditional social and economic institutions, which were branded as tools of bourgeois oppression. The subsequent Cultural Revolution (1966–1976) went further, with an ambitious goal of eradicating the “false consciousness” imposed by thousands of years of oppressive culture. Though these catastrophic policies were eventually abandoned, they severely eroded public faith in communist ideals, clearing the way for both new socioeconomic structures and a new popular culture.

The “open and reform” policies introduced in the early 1980s led to the emergence of state capitalism, characterized by aggressive privatization coupled with strict political control. Communist ideals yielded to pragmatic wealth-building. Market logic, combined with political restrictions, systematically channeled individual pursuits toward material well-being. Without facing significant counter-balancing forces from either traditional cultural institutions or the communist virtue of austerity and self-sacrifice, the long-suppressed desire to own and to prosper returned with a vengeance.

In just a few decades, Chinese society leaped from traditional agriculture through dramatic social engineering directly into prosperous, individualized modernity filled with transient experiences. With little solid ground to fall back on, people's pursuit of self-determination has metamorphosed into a quest for material possessions. This subtle transformation was vividly encapsulated in the trending term “freedom of wealth” (财富自由, *caifu ziyou*). One is reminded of Marcuse's observation of Western societies in the 1960s, which seems equally relevant to contemporary Chinese society: *people recognize themselves in their commodities and find their soul in their possessions*.

The current generation of young parents in China is the first post-reform generation, having lived through a period of significant social transformation marked by rapid marketization, the growth of the private sector, and a cultural shift toward consumerism. As materialistic values permeated social norms during their formative years, China simultaneously experienced surges in mental health issues (24, 25). The materialistic value, embraced and practiced by many (if not all) of today's young parents, have the potential to influence the wellbeing of the current children population. Growing up within this materialistic culture, the current generation of children may internalize these values and perpetuate their negative impacts across future generations. Against this social backdrop, there is an urgent need to study intergenerational health impacts of materialism and associated mechanisms. Given the hegemonic cultural status of materialism worldwide, insights from such a study will not only inform interventions in China but also guide similar efforts in other countries.

## Theory and hypothesis

Drawing on the stress process model and the life course perspective, this study developed and tested an intergenerational crossover model for materialism. According to the stress process model, stressors refer to “*conditions of threat, challenge, demands, or structural constraints that, by the very fact of their occurrence or existence, call into question the operating integrity of the organism*”. (26, p. 300). The value orientation of materialism, given its persistent nature and known threats to mental well-being, can be conceptualized as a macro-level chronic stressor capable of inducing further stressors through stress proliferation (27, 28). This proliferation occurs not only within individuals but also across connected lives, echoing the “linked lives” concept of the life course perspective (28–30). In other words, beliefs and behaviors of an individual can have ripple effects on the lives of connected others. In this framework, a parent's materialistic values constitute a stressor that can have a crossover effect on a child's life, triggering three interconnected secondary stressors: the child's materialism, a strained family relationship, and the parent's social comparison of the child.

First, an obvious pathway through which parental materialism influences child psychological well-being is the *intergenerational transmission of values* (31). Materialistic values tend to be inherited by the next generation (32–34). Parents are the most important agents of socialization, serving as the primary models from which children learn behaviors, attitudes, and values. Through observation and interaction, children absorb and internalize their parents' materialistic values, often adopting these values in defining their own benchmarks for success and happiness. This process of value transmission is greatly facilitated by the broader consumer culture that both parents and children are immersed in.

As previously mentioned, elevated materialism consistently predicts poorer psychological well-being. When these materialistic values are passed on to a child, they can jeopardize the child's mental well-being in much the same way they jeopardize the parent's well-being. A materialistically oriented individual is *growth-minded, performance-oriented, and other-directed*. A life dedicated to acquisition of material goods inevitably exposes the individual to constant uncertainties in material quest, insatiable cravings for more, a perpetual state of unsettledness, an externally contingent self-worth, a subtle urge for social comparison, and at times, a sense of relative deprivation, all of which are detrimental to one's psychological well-being. Moreover, materialistic values by nature promote extrinsic pursuits, which are less satisfying and crowd out the more rewarding and satisfying intrinsic pursuits (35). As such, I hypothesize that *higher parent materialism is associated with higher child psychological distress through increased child materialism*.

The second mechanism through which parental materialism influences a child's psychological wellbeing is the *compromised family relationship*. An intimate and harmonious family environment (including both parent-child and parent-parent relationships) is crucial for children's psychological well-being. However, materialism as a self-oriented value is incongruent with collective-oriented values such as family value, which attaches importance to close and caring family relations as a source of life purpose and meaning (36). The encroachment of materialistic values into family life could thus negatively impact family intimacy.

For one thing, high materialism and its associated extrinsic pursuit of image, popularity, and success will compete with commitment to family relationships. The materialistic pursuit often takes the form of prioritizing work over family roles. Juliet Schor (37) has long noted that the pursuit of material wealth leads to longer working hours and less time for personal relationships and community engagement. For parents, the constant pursuit of material success necessarily interferes with their family roles and erodes the quality and quantity of meaningful interactions within the family (38). Indeed, highly materialistic individuals are more likely to experience work overload and a high level of work-family conflicts (39).

For another, materialism can lead the person to interpret the well-being of family members in material terms and neglect their intrinsic psychological needs (e.g., needs for autonomy, competence, and relatedness). This materialistic lens can overshadow the emotional and developmental needs of children, fostering an environment in which emotional connections among family members are undervalued. For example, studies have found that high materialism is associated with lower qualities in marriage and in parent-child relationships (40, 41). For children, a compromised family relationship would translate into compromised mental well-being. Therefore, I hypothesize that *higher parent materialism is associated with higher child psychological distress through decreased quality of family relationships*.

Finally, parent materialism can threaten a child's psychological well-being through a *parent's social comparison of the child*, which means that the parent constantly compares his/her child with other children in their attributes (e.g., skills, appearance, personality, character, etc.). The statement that “*comparison is the thief of joy*” underscores a core principle of social comparison theory, according to which the derivation of self-evaluation through comparing the self to others could trigger feelings of inadequacy, jealousy, or inferiority (42).

Materialism has an inherent competitive nature and thus is inextricably linked with a strong social comparison orientation and an other-directed social character (9, 43, 44). People with high materialism are more concerned with approval, acceptance, and feedback from others and have strong desires to present a public image that invites envy and admiration, which inevitably requires explicitly or implicitly comparing what they have with what others have. Social comparison, however, may not necessarily be limited to oneself, but can extend those related to the self, especially when the self takes an *owner's* perspective and turns the related individuals into *possessed objects* whose values are evaluable and comparable (45). To high-materialism people, *things* they have not only include goods or products, but also people in their social networks. The stronger the social tie is, the more likely the linked person (e.g., children or wife/husband) is to be considered a *possession*, and the more likely for the social comparison to be extended to the linked person. The term “trophy wife” presents a vivid case illustrating the intrinsic link between materialism, objectification, and social comparison. I argue that a similar process could happen to children. Parents with high materialistic values tend to instrumentalize their children as social accessories or decorative pieces, whose worth is evaluated and compared in a personality market. Parents' constant social comparison of their children not only puts their own well-being at jeopardy, but also threatens the children's psychological well-being by creating constant stress for measuring up. As such, I hypothesize that *higher parental materialism is associated with higher child psychological distress* via *more frequent parental social comparison of the child*.

## Methods

This study aims to test an intergenerational crossover model that explains how parental materialism is associated with child psychological distress through three key mechanisms: (1) the transmission of materialistic values to children, (2) the deterioration of family relationship quality, and (3) increased parental social comparison of the child. I hypothesize that each of these pathways will mediate the relationship between parental materialism and child psychological distress. Specifically, higher parental materialism is expected to be associated with greater child materialism, lower family relationship quality, and more frequent social comparison, each of which will, in turn, be associated with greater psychological distress among children. To test these hypotheses, I use dyadic survey data collected from a large sample of seventh-grade students and their parents in a rapidly developing metropolitan region in China. The following sections describe the data collection procedures, sample characteristics, and the measurement of all focal constructs, including the mediators and outcome variables.

### Data

Between November and December 2016, a cross-sectional survey was conducted involving 7th grade students currently enrolled in six randomly selected middle schools in metropolitan Zhengzhou, a fast-developing provincial capital city in Central China with a population of 9.5 million. All 7th grade students currently enrolled in these six schools, along with their parents, were invited to participate. Each participating student received a survey package that included a parent questionnaire, a student questionnaire, written instructions, and standard envelopes for separately sealing completed parent/student questionnaires. Students and one of their parents (selected on a voluntary basis) were requested to complete and seal their respective questionnaires. Students brought back to school completed questionnaires in sealed envelopes and submitted them to the data collection team with assistance from the host classroom teacher. Parent-child dyadic data were constructed by linking the student and parent questionnaires. The survey was jointly administered by the Institute of Survey and Data Analytics at Zhengzhou University in collaboration with the Department of Education of Zhengzhou Municipal Government.

Out of a total of 2,320 7th grade students in the sampled schools, 2,262 participated in the study. Among these, we excluded 62 students who were not living with any parents and thus had no parental responses. Among the participating parents, 967 identified as the child's father, while 1,209 identified as the mother. Additionally, 24 cases involved individuals who identified as non-parental figures or did not disclose their parental role; these cases were excluded. Moreover, individuals with divorced/deceased parents (180 cases) were excluded, given that the measures for family relations for these individuals were inconsistent with those with both parents. Following these exclusions, the final sample size for analysis consisted of 1,996 parent-child dyads.

## Measurements

### Outcome

Child psychological distress was measured as the factor score of a 6-item scale adapted from the student questionnaire of *China Educational Panel Survey*. This scale asked the respondent to report how often during the past 30 days they have felt: *down or depressed, unhappy, so sad that nothing could cheer up, little interest in doing things, so nervous that nothing could calm down,* and *everything an effort.* Response ranged from 1 (“Never”) to 5 (“Always”). The scale displayed strong internal consistency (alpha = .88).

### Exposure

Parent materialism was measured with an 8-item materialism scale that was adapted from the materialism scale developed by Richins and Dawson (9). The scale asked the parent to rate how much they agree or disagree with the nine statements about one's materialistic value orientation (alpha = .82). Example statements include “*The things I own say a lot about how well I'm doing in life*”, “*Some of the most important achievements in life include acquiring material possessions*”, and “*I'd be happier if I could afford to buy more things*”. Response ranges from 1 (“Strongly Disagree”) to 5 (“Strongly Agree”). Supplementary Table 1 lists all the statements. Following previous practices, we recoded the factor score of the 9 items into *top quartile*, *bottom quartile*, and *in-between quartiles* and focused on comparing the low and top quartiles to achieve a clearer pattern of distinction (9, 46, 47). As a sensitivity analysis, parental materialism was alternatively modeled as a latent continuous factor score using the above indicators to test the robustness of results.

### Mediators

Child materialism was measured as the factor score of a 7-item youth-version materialism scale. The scale asked the child to rate how true the seven statements are in describing him/herself (alpha = .82). Example statements include “*I like to buy things my friends have*”, “*when I grow up, the more money I have, the happier I will be*”, and “*I'd be happier if I could afford to buy more things*”. Responses range from 1 (“Absolutely Untrue”) to 6 (“Absolutely True”). Supplementary Table S1 lists all the items.

Family relationship was measured as the factor score of an 8-item global relation scale that asked the children to rate how true the nine statements are in describing their family relationship (alpha = .86). Example statements include “*I can share feelings with my parents*”, “o*verall, I have a good relationship with my father/mother*”, and “*my parents get along very well*”. Responses range from 1 (“Absolutely Untrue”) to 6 (“Absolutely True”). Supplementary Table S1 lists all the items.

Parents' social comparison of child was measured as the factor score of a 7-item scale that asked the child to report how often their parents draw comparisons between him/her and other children in the following aspects: academic performance, obedience to parents, artistic talents, intelligence, appearance, accountability, and willingness to help. Responses ranged from 1 (“Never”) to 6 (“Almost every day”). The scale displayed strong internal consistency (alpha = .80).

### Covariates

The analysis adjusted for the following covariates: child age (in years), child sex (male, female), *hukou* (urban, rural), family income (annual income <¥50k, ¥50k≤ annual income <¥100k, ¥100k≤ annual income <¥150k, ¥150k≤ annual income), single child status (yes, no), responding parent education (middle school or below, high school, college or above), and responding parent occupation (non-manual professional, skilled/non-skilled manual labor, small business owner, unemployed).

### Analytic strategy

I first presented descriptive statistics for the full analytic sample, as well as for subsamples stratified by quartiles of parental materialism scores (i.e., top, bottom, and in-between). I conducted chi-squared tests (for categorical variables) and *F*-tests (for continuous variables) to identify significant disparities in sample characteristics across different levels of parent materialism. In addition to testing overall group differences using ANOVA, I conducted pairwise *post hoc* comparisons to identify which specific group contrasts drove the observed effects. Both Bonferroni and Tukey adjustments were applied to adjust for multiple comparisons. To assess the practical significance of the findings, I calculated partial eta-squared for each outcome variable and Cohen's d for all pairwise group differences.

**Figure 1:**
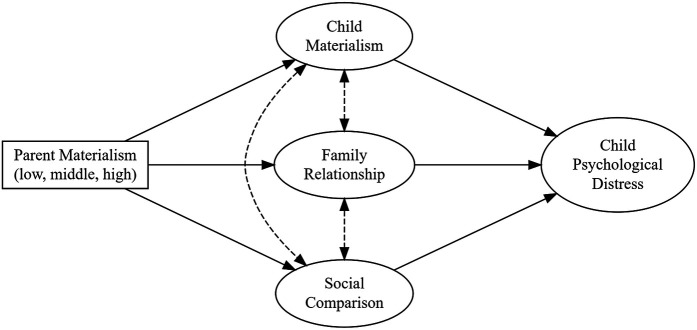
The structural equation model for the intergenerational stress crossover of parent materialism.

To test the hypotheses, I fitted a structural equation model (SEM) that includes measurement models for mediators and the outcome, along with a structural model in which parent materialism predicts child psychological distress through child materialism, family relationship, and parent social comparison of child. All mediators were simultaneously included in a single unified SEM, with their residual variances allowed to correlate to reflect empirical interdependencies. Parent materialism was included as a categorical observed variable due to its operationalization based on quartiles, consistent with prior studies. While this approach aids interpretability, it does not account for measurement error in the exogenous variable, which may affect precision. To assess the robustness of this specification, I conducted a sensitivity analysis using the continuous latent factor score of parental materialism. All regression paths in the SEM were adjusted for the full set of covariates. Figure 1 presents the model structure. A detailed introduction of the SEM specification is described below.

The measurement component of the SEM can be expressed in matrix form as:

y=Λη+ϵ

Where: y is the vector of all observed indicators (items) for the latent variables; $\eta =[\eta_{1},\eta_{2},\eta_{3},\eta_{4}]$ is the vector of latent constructs, where $\;\eta_{1}$ to $\eta_{4}$ respectively represent Child Materialism, Family Relationship Quality, Parental Social Comparison of Child, and Child Psychological Distress; Λ is the matrix of factor loadings that maps the latent variables onto their observed indicators; ϵ is the vector of measurement errors for each observed indicator, assumed to be uncorrelated within constructs.

The structural component of the SEM can be expressed as:

$$\begin{array}{rl} & \eta_{1}=\gamma_{10}+\gamma_{11}\mathrm{Parental}\;\mathrm{Materialism}\;+\Gamma_{1}C+\zeta_{1}\\ & \eta_{2}=\gamma_{20}+\gamma_{21}\mathrm{Parental}\;\mathrm{Materialism}\;+\Gamma_{2}C+\zeta_{2}\\ & \eta_{3}=\gamma_{30}+\gamma_{31}\mathrm{Parental}\;\mathrm{Materialism}\;+\Gamma_{3}C+\zeta_{3}\\ & \eta_{4}=\gamma_{40}+\beta_{1}\eta_{1}+\beta_{2}\eta_{2}+\beta_{3}\eta_{3}+\beta_{4}\mathrm{Parental}\;\mathrm{Materialism}+\Gamma_{4}4C+\zeta_{4} \end{array}$$

Where: $\eta_{1}$ to $\eta_{4}$ respectively represent Child Materialism, Family Relationship Quality, Parental Social Comparison of Child, and Child Psychological Distress; $\gamma_{i1}$ represents the coefficient for parental materialism in predicting $\eta_{i}$; $\beta_{1-3}$ are path coefficients from mediators to child psychological distress ($\eta_{4}$) while $\beta_{4}$ is the direct path coefficient from parental materialism to child psychological distress; C is a vector of covariates with coefficients Γ; residual covariances among $\zeta_{1},\;\zeta_{2},\;\zeta_{3}$ were freely estimated to account for interdependencies among mediators.

Model estimation used maximum likelihood with robust standard errors (MLR). Around 25% of observations have missing values in at least one variable. Missing data were addressed via Full Information Maximum Likelihood (FIML), which maximizes the use of available data under the missing at random (MAR) assumption. Sensitivity analyses using listwise deletion were also performed to test results robustness. Standard model fit indices (RMSEA, CFI, TLI, SRMR) were reported.

## Results

Table 1 presents the sample characteristics stratified by levels of parental materialism (lowest, highest, and in-between quartiles). ANOVA results reveal significant differences in child psychological distress, child materialism, family relationship quality, and parent social comparison of child across the levels of parental materialism. Children from families in the highest quartile of parental materialism report significantly higher levels of psychological distress compared to those in the lowest quartile (*p* < 0.01). Similarly, children's materialism scores are highest among those with parents in the top quartile of materialism and lowest among those in the bottom quartile (*p* < 0.001).

**Table 1:** Sample characteristics (mean ± sd or %) by levels of parental materialism (*N* = 1,996).

Variables	Parental materialism			Total	*P*-value^a^
	Lowest quartile	In-between quartiles	Highest quartile		
Child psychological distress^b^	−0.13 ± .99	−0.00 ± .97	0.07 ± 1.00	−0.02 ± .98	**0** **.** **009**
Child materialism^b^	−0.24 ± .88	0.01 ± .97	0.25 ± 1.07	0.00 ± .99	**0** **.** **000**
Family relationship quality^b^	−0.09 ± 1.02	−0.05 ± .99	0.20 ± .96	0.00 ± 1.00	**0** **.** **000**
Parent social comparison of child^b^	2.35 ± .86	2.45 ± .82	2.59 ± .83	2.46 ± .84	**0** **.** **000**
Age	12.48 ± .64	12.45 ± .85	12.48 ± .59	12.46 ± .74	0.659
Male	50.00	52.85	58.01	53.33	**0** **.** **046**
Rural Hukou	59.03	57.82	64.64	59.79	**0** **.** **050**
Only child in family	11.47	12.83	11.49	12.14	0.695
Responding parent					
Father	42.54	45.57	54.69	46.97	**0** **.** **000**
Mother	57.46	54.43	45.31	53.03	
Responding parent education					
Middle school or below	62.24	59.89	61.28	60.86	0.351
High school	28.98	28.60	26.20	28.13	
College or above	8.78	11.51	12.53	11.02	
Family income (in RMB)					
<50,000	42.53	39.66	46.70	42.15	**0** **.** **031**
50,000–100,000	39.16	41.37	33.03	38.74	
100,000–150,000	9.05	11.77	10.48	10.73	
>150,000	9.26	7.20	9.79	8.38	
Responding parent occupation					
Non-manual	22.87	18.22	21.65	20.30	0.078
Manual labor	33.60	38.78	40.63	37.84	
Small business owner	26.11	26.78	25.00	26.17	
Unemployed	17.41	16.22	12.72	15.69	

Boldface indicates statistical significance.

^a^ *p*-values are from chi-square tests for categorical variables and *F*-tests for continuous variables.

^b^ Factor scores.

Additionally, the quality of family relationships shows a negative association with levels of parental materialism, with children from the highest materialism quartile reporting the lowest family relationship quality compared to the lowest quartile (*p* < 0.001). Parent social comparison of child scores also varies significantly, being highest in the highest materialism quartile and lowest in the lowest quartile (*p* < 0.001).

Post hoc analyses indicate that these significant group differences are primarily driven by contrasts between the highest and lowest quartiles of parental materialism. For example, in child materialism, the difference between the lowest and highest quartiles was substantial (Cohen's *d* = .50), while comparisons involving the in-between group showed smaller effects (*d* = .27 and *d* = .23). Similar patterns emerged for psychological distress, family relationship quality, and parental social comparison, with the largest differences consistently observed between the extremes of parental materialism. To further assess practical significance, partial eta-squared was computed for each outcome. Parental materialism explained 4.7% of the variance in child materialism, 3.4% in family relationship quality, 3.7% in parental social comparison, and 1.8% in psychological distress, indicating small to moderate effect sizes.

Sociodemographic variables such as age, gender, hukou status, only child status, and responding parent's education show no significant disparities across different levels of parental materialism. Fathers were more likely to report high materialism than mothers; those with rural *hukou* were more likely to report high materialism than those with urban hukou. Those whose annual family income fell at the bottom (i.e., <50k) tend to be overrepresented in the highest parental materialism quartile.

Table 2 reports the standardized regression coefficients and model fit statistics for the mediation analysis. The SEM demonstrated good model fit: RMSEA = 0.033 [90% CI: 0.031–0.034, p(RMSEA ≤0.05) = 1.000]; CFI = 0.930; TLI = 0.922; SRMR = 0.032. The excellent RMSEA and SRMR, along with acceptable CFI and TLI, indicate that the model adequately captures the data's covariance structure. The results also identified several statistically significant pathways linking parental materialism to child psychological distress. In comparison to the lowest quartile of parental materialism, the highest quartile of parental materialism was associated with higher level of child materialism (*β* = 0.60, *p* < 0.001), lower family relationship quality (*β* = −0.34, *p* < 0.001), and higher parental social comparison of the child (*β* = 0.36, *p* < 0.001). Child materialism (*β* = 0.13, *p* < 0.001) and parent social comparison (*β* = 0.24, *p* < 0.001) positively predict child psychological distress, while family relationship quality negatively predicts it (*β* = −0.29, *p* < 0.001). The total indirect effects of high parental materialism on child psychological distress through these mediators are significant (*β* = 0.26, *p* < 0.001). After accounting for these three mediators, parental materialism was no longer associated with child psychological distress, suggesting that these three mechanisms completely explained the intergenerational association between parental materialism and child psychological distress. Decomposition of the indirect effects suggested that the three pathways carry similar weights. The pathway from high parent materialism to child psychological distress through child materialism is significant (*β* = 0.08, *p* < 0.001). Similarly, the pathways through family relationship quality (*β* = 0.10, *p* < 0.001) and parent social comparison (*β* = 0.09, *p* < 0.001) are also significant. Although the cross-sectional study cannot determine causality, these significant pathways provided some suggestive evidence for the intergenerational link between parental materialism to child mental health.

**Table 2:** Standardized regression coefficients (95% confidence intervals) from the intergenerational crossover model for parental materialism (*N* = 1,996).

Predictors	Mediators			Child psychological distress
	Child materialism	Family relationship	Parent social comparison of child	
Parent materialism				
Low	Reference			
Middle	**0.30** ***	**−0.15** **	**0.14** *	0.04
	**[0.19, 0.41]**	[−0.27, −0.04]	[0.01, 0.26]	[−0.08, 0.16]
High	**0.60** ***	**−0.34** ***	**0.36** ***	−0.03
	**[0.45, 0.74]**	**[−0.48, −0.19]**	**[0.22, 0.51]**	[−0.18, 0.12]
Child materialism				**0.13** ***
				**[0.07, 0.20]**
Family relationship				**−0.29** ***
				**[−0.36, −0.22]**
Parent social comparison of child				**0.24** ***
				**[0.17, 0.31]**
Age	0.02	−0.03	−0.02	−0.03
	[−0.07, 0.11]	[−0.09, 0.03]	[−0.11, 0.08]	[−0.13, 0.07]
Male	**0.14** ***	**−0.10** **	**0.22** ***	**−0.30** ***
	**[0.04, 0.24]**	**[−0.20, −0.01]**	**[0.11, 0.32]**	**[−0.40, −0.20]**
Rural Hukou	0.09	0.04	**0.17** **	0.03
	[−0.01, 0.19]	[−0.07, 0.14]	**[0.06, 0.28]**	[−0.08, 0.13]
Only Child In Family	−0.03	0.1	0.02	0.06
	[−0.20, 0.14]	[−0.07, 0.27]	[−0.15, 0.19]	[−0.12, 0.24]
Responding parent: mother	0.04	**−0.10** *	−0.08	0.05
	[−0.06, 0.14]	**[−0.20, −0.00]**	[−0.19, 0.03]	[−0.05, 0.16]
Responding parent education				
Middle school or below	Reference			
High school	−0.08	**0.17** **	**−0.12** *	0.03
	[−0.20, 0.03]	**[0.05, 0.29]**	**[−0.24, 0.00]**	[0.03, 0.14]
College or above	−0.20	**0.25** **	−0.17	0.13
	[−0.38, −0.02]	**[0.08, 0.42]**	[−0.36, 0.02]	[0.12, 0.29]
Family income brackets				
<50,000	Reference			
50,000–100,000	−0.08	−0.13	−0.09	−0.01
	[−0.20, 0.03]	[−0.13, 0.01]	[−0.21, 0.04]	[−0.13, 0.10]
100,000–150,000	0.12	−0.09	0.04	−0.02
	[−0.07, 0.30]	[−0.09, 0.06]	[−0.15, 0.23]	[−0.19, 0.15]
>150,000	−0.09	−0.06	0.15	0.01
	[−0.28, 0.11]	[−0.06, 0.11]	[−0.07, 0.36]	[−0.20, 0.21]
Responding parent occupation				
Non-manual	Reference			
Manual labor	0.05	−0.13	−0.01	0.02
	[−0.11, 0.20]	[−0.13, 0.01]	[−0.17, 0.15]	[−0.13, 0.17]
Small business owner	0	−0.09	−0.03	0.00
	[−0.15, 0.16]	[−0.09, 0.06]	[−0.19, 0.13]	[−0.15, 0.15]
Unemployed	0.03	−0.06	0.10	−0.03
	[−0.15, 0.21]	[−0.06, 0.11]	[−0.09, 0.28]	[−0.20, 0.14]
Indirect effects				
Total indirect effects:				**0.26** ***
				**[0.19, 0.34]**
Indirect effects decomposition:				
High parent materialism → Child Materialism → Child psychological distress				**0.08** ***
				**[0.04, 0.12]**
High parent materialism → Family relationship → Child psychological distress				**0.10** ***
				**[0.05, 0.14]**
High parent materialism → Parent social comparison of child → Child psychological distress				**0.09** ***
				**[0.04, 0.13]**
Goodness-of-fit indices	Estimate	90% C.I.		
RMSEA (root mean square error of approximation)	0.033	[0.031, 0.034]		
CFI	0.930			
TLI	0.922			
SRMR (standardized root mean square residual)	0.032			

95% confidence intervals in brackets. Boldface indicates statistical significance.

* *p* < 0.05.

** *p* < 0.01.

*** *p* < 0.001.

### Sensitivity analysis

To test the robustness of results, I conducted a series of sensitivity analyses. First, I re-estimated the model using the continuous latent factor score of parental materialism as the exogenous predictor instead of the quartile-based categorical variable. Second, I repeated the analysis using listwise deletion to handle missing data, in contrast to the main analysis which used Full Information Maximum Likelihood (FIML). The results showed that the overall pattern of associations remained consistent across both alternative specifications. Using the continuous predictor produced smaller coefficients, as expected due to scale differences, but the direction and significance of key pathways were unchanged. The listwise deletion model also yielded substantively similar results. These findings support the robustness of the main conclusions. Estimates for focal relationships from these sensitivity analyses were summarized in Supplementary Table S2.

## Discussion

When Max Weber introduced the term “iron cage”, he used it to refer to the care for material goods, an internal drive that Puritans once called “*a light cloak [that] can be thrown aside at any moment*”. Sociological studies generally recognized that the “iron cage” traps individuals in a rigid, rational, and dehumanizing system. However, despite the central place of culture in sociology and the classical sociological discussions of materialism, there are few middle-range theories to explain how the “iron cage” forged by one generation harms the health of the next. As such, little advice was given regarding interventions, adding yet another tint of inescapability to the “iron cage” thesis.

The intergenerational crossover model I proposed offered a glimpse into how this “iron cage” may create intergenerational health links. It outlined three mechanisms through which parent materialism predicts child mental health: intergenerational transmission of materialistic values, compromised family relations, and parent's social comparison of the child. Consistent with its hypotheses, the study found that higher levels of parental materialism were linked to increased materialistic values in children, diminished family relationship quality, and more frequent parental social comparisons of the child. These elements together are associated with higher levels of psychological distress among children. Although the effect sizes detected in this study were small to moderate, they are consistent with prior research on psychosocial stressors and reflect meaningful public health implications when considered at the population level. The evidence for these significant pathways thus provided a roadmap for future youth mental health interventions.

Although this study was conducted in China, these findings echo research from Western societies where materialism is linked to poor mental health, weak family ties, externalized self-worth, loneliness, addictive behaviors, and social dysfunction (7, 14–18). This suggests that the interpersonal crossover risks of materialistic orientation may transcend cultural boundaries, especially under global consumer capitalism. I thus argue that the model could be applied to study the generational health impact of materialistic values in modern consumer societies at large as well as societies going through rapid modernization.

Materialism could be consideredthe “Default Value of Modernity”, haunting all modern societies and those aspiring for the modernization agenda. This framing of “default” draws on Mirowsky and Ross's concept of the “default American lifestyle”, which describes a standard unhealthy mode of life in today's US (48). A “default” mode is what an individual naturally adopts unless intentionally overridden through agentic choices. Given the unhealthy default, health therefore depends on the individual ability to resist it and find alternatives, which is inextricably tied to the person's social position. A parallel can be drawn regarding materialism as the “default value of modernity”. In today's market-driven societies, materialism is an omnipresent cultural orientation that individuals are born into. It represents a persistent and hegemonic cultural element that is deeply rooted in a set of institutional and cultural conditions. Institutionally, growth-driven and spending-stimulating policies, ubiquitous, transactional actions and consumerism cues, media saturation, and practices of planned obsolescence created a strong institutional foundation for the dominance of materialism in the value system. Culturally, as the trend towards individualization intensifies, there emerges a need for a value system capable of redirecting modern individual's quest for autonomy and self-fulfillment towards a form that is symbiotic and collaborative with the capitalist economy, rather than antagonistic or revolutionary (49). The cultural gravitation towards materialism and consumer capitalism perfectly served for this purpose by turning the “autonomous self”, a cherished ideal since the Enlightenment, into the “autonomous consumer”, whose intrinsic worth and dignity were supplanted by the freedom to choose what to own or consume (50–52). This cultural shift to materialism satisfied the mass desire for freedom and control, not despite capitalism but through it, albeit with the cheaper substitute of commodities.

Materialism, as a pervasive “default” in modern consumer societies, can only be mitigated through intentional and agentic choices. This positions materialism as a significant contextual factor in the intergenerational transmission of inequalities, given that the resources required to make such overriding choices are unevenly distributed across families. Despite its potential impact on family wellbeing, the default nature of materialistic culture has led many scholars to overlook its significance—perhaps because it is too ingrained to be recognized or perhaps, we are buying in too much the Weberian sense of inevitability. To date, studies on family wellbeing remain surprisingly silent on this topic. Considering the omnipresence of materialistic values and their far-reaching implications for human health, future studies need to reclaim this neglected area and examine how materialism may perpetuate health inequalities across generations.

The intergenerational crossover model offers a vital starting point, not only by emphasizing the generational link between child mental health and parent materialism but also by identifying modifiable mediators that could disrupt this link. This gives hope that the generational curse of materialism is not unbreakable; rather, it can be avoided by cutting off the chain of risks through interventions in value socialization, family bonding, and parenting practices. These findings offer practical implications for intervention. For example, family-based programs can promote intrinsic parenting goals, while schools can incorporate critical value education and digital literacy curricula. At a societal level, policies that deemphasize material status competition and promote alternative well-being indicators may help buffer children from these psychosocial stressors. I hope the intergenerational crossover model can inspire further conversations by highlighting these actionable areas.

While this manuscript critically examined the negative side of materialism, I acknowledge that such a portrayal may appear overly one-sided without cultural qualification. The social meanings and psychological consequences of materialism are embedded in specific cultural and economic contexts. In societies or subcultures where neoliberal ideologies are dominant, or where material success is tightly linked to personal empowerment, materialism may function as a form of motivational capital rather than pathology. For example, among precarious families experiencing upward mobility, it can serve as an identity marker for competence, care, or resilience. Accordingly, materialism may elicit pride, hope, and ambition, rather than distress. The impact of materialism on wellbeing is thus likely contingent upon intersecting factors such as economic security, cultural values, and broader parenting ideologies. These possibilities suggest the importance of examining potential moderators that may condition the effects of materialism across different social groups. Future analyses should explore how economic situations, family norms, and other contextual factors may shape these pathways, allowing for a more nuanced and context-sensitive understanding of intergenerational processes.

This study is not without its limitations. First, the sample was drawn from a single metropolitan area in China, which limits the generalizability of the findings to rural areas and other regions. Future replication studies should incorporate diverse populations across different sociocultural contexts to enhance generalizability. Second, given the cross-sectional nature of the data, findings should be interpreted as correlational rather than causal. Although SEM paths are specified based on theory, the model cannot determine time order. Bidirectional or unmeasured confounding relationships may exist. For instance, children's distress may lead to compensatory materialism in parents, and strained family functioning could be both a cause and consequence of parental materialism. Longitudinal or experimental designs are needed to clarify the causal structure of the observed associations and/or test these potentially reciprocal pathways. Third, the data used in this study were collected in 2016. While materialism's structural and psychological logic likely persists, some cultural and economic contexts may have evolved. For example, pandemic-era disruptions and economic hardships may either heighten or crowd out many of the pressures discussed here. Future studies using more recent or longitudinal data are warranted. Lastly, the study's limited scope did not allow for the assessment of potential moderators in the pathways examined. I plan to address this gap in the future by investigating various moderators to gain a more comprehensive understanding of the process. Despite these limitations, this is the first sociological study proposing and testing a middle-range theory on the intergenerational stress proliferation triggered by materialism. It provided a framework and the first evidence guiding our understanding of the generational health impacts of this pervasive but understudied cultural phenomenon in modern consumer societies.

## Data Availability

The raw data supporting the conclusions of this article will be made available upon request.

## References

[1] Hidaka BH. Depression as a disease of modernity: explanations for increasing prevalence. J Affect Disord. (2012) 140(3):205–14. 10.1016/j.jad.2011.12.036 22244375 PMC3330161

[2] Okasha A. Globalization and mental health: a WPA perspective. World Psychiatry. (2005) 4(1):1–2. 16633493 PMC1414710

[3] Schumaker JF. The Age of Insanity: Modernity and Mental Health. Westport, CT: Praeger Publisher (2001).

[4] Westberg KH Nyholm M Nygren JM Svedberg P. Mental health problems among young people-a scoping review of help-seeking. Int J Environ Res Public Health. (2022) 19(3):1–15. 10.3390/ijerph19031430 PMC8835517 35162452

[5] Collishaw S Sellers R. Trends in child and adolescent mental health prevalence, outcomes, and inequalities. In: Taylor E Verhulst F Wong JCM Yoshida K, editors. Mental Health and Illness of Children and Adolescents. Singapore: Springer (2020). p. 63–73. 10.1007/978-981-10-2348-4_9

[6] Shorey S Ng ED Wong CHJ. Global prevalence of depression and elevated depressive symptoms among adolescents: a systematic review and meta-analysis. Br J Clin Psychol. (2022) 61(2):287–305. 10.1111/bjc.12333 34569066

[7] Dittmar H Bond R Hurst M Kasser T. The relationship between materialism and personal well-being: a meta-analysis. J Pers Soc Psychol. (2014) 107(5):879–924. 10.1037/a0037409 25347131

[8] Kasser T. Materialistic values and goals. Annu Rev Psychol. (2016) 67:489–514. 10.1146/annurev-psych-122414-033344 26273896

[9] Richins ML Dawson S. A consumer values orientation for materialism and its measurement—scale development and validation. J Consum Res. (1992) 19(3):303–16. 10.1086/209304

[10] Engelberg E Sjoberg L. Money attitudes and emotional intelligence. J Appl Soc Psychol. (2006) 36(8):2027–47. 10.1111/j.0021-9029.2006.00092.x

[11] Ger G Belk RW. Cross-cultural differences in materialism. J Econ Psychol. (1996) 17(1):55–77. 10.1016/0167-4870(95)00035-6

[12] Auerbach RP McWhinnie CM Goldfinger M Abela JRZ Zhu X Yao S. The cost of materialism in a collectivistic culture: predicting risky behavior engagement in Chinese adolescents. J Clin Child Adolesc Psychol. (2010) 39(1):117–27. 10.1080/15374410903401179 20390803

[13] Bernardo ABI Tan-Mansukhani R Daganzo MAA. Associations between materialism, gratitude, and well-being in children of overseas Filipino workers. Eur J Psychol. (2018) 14(3):581–98. 10.5964/ejop.v14i3.1555 30263072 PMC6143983

[14] Lipovcan LK Prizmic-Larsen Z Brkljacic T. Materialism, affective states, and life satisfaction: case of Croatia. Springerplus. (2015) 4:699. 10.1186/s40064-015-1494-5 26587367 PMC4644134

[15] Maras P Moon A Gupta T Gridley N. The role of materialism on social, emotional and behavioural difficulties for British adolescents. Emot Behav Diffic. (2015) 20(4):362–80. 10.1080/13632752.2014.989055

[16] Muniz-Velazquez JA Gomez-Baya D Lopez-Casquete M. Implicit and explicit assessment of materialism: associations with happiness and depression. Pers Individ Dif. (2017) 116:123–32. 10.1016/j.paid.2017.04.033

[17] Roberts JA Tsang J-A Manolis C. Looking for happiness in all the wrong places: the moderating role of gratitude and affect in the materialism-life satisfaction relationship. J Pos Psychol. (2015) 10(6):489–98. 10.1080/17439760.2015.1004553

[18] Wang R Liu H Jiang J Song Y. Will materialism lead to happiness? A longitudinal analysis of the mediating role of psychological needs satisfaction. Pers Individ Dif. (2017) 105:312–7. 10.1016/j.paid.2016.10.014

[19] Brown KW Kasser T. Are psychological and ecological well-being compatible? The role of values, mindfulness, and lifestyle. Soc Indic Res. (2005) 74(2):349–68. 10.1007/s11205-004-8207-8

[20] Kasser T. Teaching about values and goals: applications of the circumplex model to motivation, well-being, and prosocial behavior. Teach Psychol. (2014) 41(4):365–71. 10.1177/0098628314549714

[21] Koo J. Long-term relationship among materialism, interpersonal relations, and happiness [청소년기 물질주의와 대인관계의 질, 성인기 행복의 장기적 관계]. Korean J Youth Stud. (2018) 25(6):169–89. 10.21509/kjys.2018.06.25.6.169

[22] Pieters R. Bidirectional dynamics of materialism and loneliness: not just a vicious cycle. J Consum Res. (2013) 40(4):615–31. 10.1086/671564

[23] Yang Z Fu X Yu X Lv Y. Longitudinal relations between adolescents’ materialism and prosocial behavior toward family, friends, and strangers. J Adolesc. (2018) 62:162–70. 10.1016/j.adolescence.2017.11.013 29197702

[24] Jiang J. Anxiety from the perspective of modernity: an empirical study based on the Chinese general social survey of 2005–2013. J Health Psychol. (2020) 25(8):1138–52. 10.1177/1359105318819052 30565489

[25] Sun J Ryder AG. The Chinese experience of rapid modernization: sociocultural changes, psychological consequences? Front Psychol. (2016) 7(477):1–13. 10.3389/fpsyg.2016.00477 27092093 PMC4820454

[26] Wheaton B Young M Montazer S Stuart-Lahman K. Social stress in the twenty-first century. In: Aneshensel CS Phelan JC Bierman A, editors. Handbook of the Sociology of Mental Health. Netherlands: Springer (2013). p. 299–323. 10.1007/978-94-007-4276-5_15

[27] Pearlin LI. The stress process revisited: reflections on concepts and their interrelationships. In: Aneshensel CS Phelan JC, editors. Handbook of the Sociology of Mental Health. New York, NY: Kluwer Academic/Plenum (1999). p. 395–415.

[28] Pearlin LI Schieman S Fazio EM Meersman SC. Stress, health, and the life course: some conceptual perspectives. J Health Soc Behav. (2005) 46(2):205–19. 10.1177/002214650504600206 16028458

[29] Thoits PA. Stress and health: major findings and policy implications. J Health Soc Behav. (2010) 51(1 suppl):S41–53. 10.1177/0022146510383499 20943582

[30] Turney K. Stress proliferation across generations? Examining the relationship between parental incarceration and childhood health. J Health Soc Behav. (2014) 55(3):302–19. 10.1177/0022146514544173 25138199

[31] Min J Silverstein M Lendon JP. Intergenerational transmission of values over the family life course. Adv Life Course Res. (2012) 17(3):112–20. 10.1016/j.alcr.2012.05.001

[32] Richins ML Chaplin LN. Material parenting: how the use of goods in parenting fosters materialism in the next generation. J Consum Res. (2015) 41(6):1333–57. 10.1086/680087

[33] Russell CA Shrum LJ. The cultivation of parent and child materialism: a parent-child dyadic study. Hum Commun Res. (2021) 47(3):284–308. 10.1093/hcr/hqab004 34248418 PMC8252968

[34] Zawadzka AM Kasser T Borchet J Iwanowska M Lewandowska-Walter A. The effect of materialistic social models on teenagers’ materialistic aspirations: results from priming experiments. Curr Psychol. (2021) 40(12):5958–71. 10.1007/s12144-019-00531-3

[35] Sheldon KM Ryan RM Deci EL Kasser T. The independent effects of goal contents and motives on well-being: it’s both what you pursue and why you pursue it. Pers Soc Psychol Bull. (2004) 30(4):475–86. 10.1177/0146167203261883 15070476

[36] Burroughs J Xa E Rindfleisch A. Materialism and wellbeing: a conflicting values perspective. J Consum Res. (2002) 29(3):348–70. 10.1086/344429

[37] Schor JB. The Overworked American: The Unexpected Decline of Leisure. New York, NY: Basic Books (1991).

[38] Pugh AJ. The Tumbleweed Society: Working and Caring in an Age of Insecurity. New York, NY: Oxford University Press (2015).

[39] Promislo MD Deckop JR Giacalone RA Jurkiewicz CL. Valuing money more than people: the effects of materialism on work–family conflict. J Occup Organ Psychol. (2010) 83(4):935–53. 10.1348/096317909X480167

[40] Allsop DB Wang C-Y Dew JP Holmes EK Hill EJ Leavitt CE. Daddy, mommy, and money: the association between parental materialism on parent-child relationship quality. J Fam Econ Issues. (2021) 42(2):325–34. 10.1007/s10834-020-09705-9

[41] Carroll JS Dean LR Call LL Busby DM. Materialism and marriage: couple profiles of congruent and incongruent spouses. J Couple Relatsh Ther. (2011) 10(4):287–308. 10.1080/15332691.2011.613306

[42] Hu Y Zhou M Shao Y Wei J Li Z Xu S The effects of social comparison and depressive mood on adolescent social decision-making. BMC Psychiatry. (2021) 21(1):3. 10.1186/s12888-020-02928-y 33402153 PMC7786518

[43] Belk RW. Materialism: trait aspects of living in the material world. J Consum Res. (1985) 12(3):265–80. 10.1086/208515

[44] Riesman D Denny R Glazer N. The Lonely Crowd; a Study of the Changing American Character. New Haven, CT: Yale University Press (1950).

[45] Saunders S Munro D. The construction and validation of a consumer orientation questionnaire (SCOI) designed to measure Fromm’s (1995) “marketing character” in Australia. Soc Behav Pers. (2000) 28(3):219–40. 10.2224/sbp.2000.28.3.219

[46] Goldberg ME Gorn GJ Peracchio LA Bamossy G. Understanding materialism among youth. J Consum Psychol. (2003) 13(3):278–88. 10.1207/S15327663JCP1303_09

[47] Richins ML Rudmin FW. Materialism and economic psychology. J Econ Psychol. (1994) 15(2):217–31. 10.1016/0167-4870(94)90001-9

[48] Mirowsky J Ross CE. Education, health, and the default American lifestyle. J Health Soc Behav. (2015) 56(3):297–306. 10.1177/0022146515594814 26272989

[49] Marcuse H. Counter-Revolution and Revolt. 2nd ed. Boston, MA: Beacon Press (1989).

[50] Blühdorn I. The dialectic of democracy: modernization, emancipation and the great regression. Democratization. (2020) 27(3):389–407. 10.1080/13510347.2019.1648436

[51] Moldes O Ku L. Materialistic cues make US miserable: a meta-analysis of the experimental evidence for the effects of materialism on individual and societal well-being. Psychol Mark. (2020) 37(10):1396–419. 10.1002/mar.21387

[52] Shrum LJ Chaplin LN Lowrey TM. Psychological causes, correlates, and consequences of materialism. Consum Psychol Rev. (2022) 5(1):69–86. 10.1002/arcp.1077

