# Supplementary material for: The price of possessiveness: how parental materialism undermines child psychological wellbeing
Source: Front Child Adolesc Psychiatry. 2025 Aug 7;4:1600599. doi: 10.3389/frcha.2025.1600599 (PMC12367775; doi:10.3389/frcha.2025.1600599)
Supplement: Supplementary file 1 [file Table1.pdf]

Supplemental Table 1. Survey items in measurement models

Variables	Indices	Factor Loadings
Child Psychological Distress	***How often during the past 30 days they have felt:***	
	Depressed	0.83
	Not happy	0.85
	Life meaningless	0.79
	Sad	0.83
	Anxious	0.67
	Everything an effort	0.72
Parent Materialism	***How true are the following statements in describing your family relationship:***	
	I admire people who own expensive homes, cars, and clothes	0.73
	Some of the most important achievements in life include acquiring material possessions	0.70
	The things I own say a lot about how well I'm doing in life	0.65
	Owning expensive things (e.g., cars, watches, jewelries, etc.) makes me feel honorable (you mian zi)	0.80
	Buying things gives me a lot of pleasure	0.58
	I like a lot of luxury in my life	0.63
	I'd be happier if I could afford to buy more things	0.69
	It sometimes bothers me quite a bit that I can't afford to buy all the things I'd like	0.59
Child Materialism	***How true are the following statements in describing yourself:***	
	I want to have things that my friends have	0.60
	When I grow up, I will feel happy if I am rich	0.67
	When I grow up, I will feel successful if I am rich	0.58
	I am jealous of those children with many good toys and pretty dresses.	0.78
	I am jealous of children with rich parents	0.79
	I am jealous of children with lots of pocket money	0.77
	I will feel honorable (you mian zi) if I could have an extravagant birthday party.	0.67
Family Relation	***How true are the following statements in describing your family relationship:***	
	I can share feelings with my parents and family	0.78
	I can get care and help I need from my parents and family	0.79
	I have a good relationship with my father	0.78
	I have a good relationship with my mother	0.78
	I rarely have conflicts with my family	0.68
	I'm willing to tell my parents my thoughts	0.72
	My parents have very good relationship	0.81

	My family atmosphere is generally very harmonious	0.73
Competitive Parenting	***How often do your parents draw comparisons between you and other children in the following aspects:***	
	Academic performance	0.65
	Being obedient	0.72
	Having artistic talents (e.g., music, dance, etc.)	0.67
	Intelligence	0.77
	Physical appearance	0.66
	Sense of responsibility	0.69
	Being kind and helpful to others	0.60
