# Supplementary material for: The price of possessiveness: how parental materialism undermines child psychological wellbeing
Source: Front Child Adolesc Psychiatry. 2025 Aug 7;4:1600599. doi: 10.3389/frcha.2025.1600599 (PMC12367775; doi:10.3389/frcha.2025.1600599)
Supplement: Supplementary file 2 [file Table2.pdf]

Supplemental Table 2. Standardized regression coefficients (95% confidence intervals) from sensitivity analyses.

Robustness Test 1: parent materialism measured as continuous latent factor score (N=1,996)

	Mediators			Child Psychological Distress
	Child Materialism	Family Relationship	Parent Social Comparison of Child	
Parent Materialism	**0.22*** [0.17,0.28]**	**-0.15*** [-0.20,-0.09]**	**0.17*** [0.11,0.23]**	-0.04 [-0.11,0.02]
Child Materialism				**0.14*** [0.07,0.21]**
Family Relationship				**-0.29*** [-0.36,-0.22]**
Parent Social Comparison of Child				**0.24*** [0.17,0.31]**

Robustness Test 2: listwise deleting missing data (N=1,505)

	Mediators			Child Psychological Distress
	Child Materialism	Family Relationship	Parent Social Comparison of Child	
Parent Materialism				
Low	Reference			
Middle	**0.29*** [0.19,0.41]**	**-0.17** [-0.27,-0.04]**	**0.16* [0.01,0.26]**	0.04 [-0.08,0.16]
High	**0.61*** [0.45,0.74]**	**-0.36*** [-0.48,-0.19]**	**0.43*** [0.22,0.51]**	-0.03 [-0.18,0.12]
Child Materialism				**0.12** [0.07,0.20]**
Family Relationship				**-0.29*** [-0.36,-0.22]**
Parent Social Comparison of Child				**0.23*** [0.17,0.31]**

All models adjusted for the same sets of covariates as in the main analysis;
95% confidence intervals in brackets
* p < 0.05, ** p < 0.01, *** p < 0.001
